# Perception of the healthcare professionals towards the current trauma and emergency care system in Kabul, Afghanistan: a mixed method study

**DOI:** 10.1186/s12913-020-05845-8

**Published:** 2020-10-29

**Authors:** Umerdad Khudadad, Wafa Aftab, Asrar Ali, Nadeem Ullah Khan, Junaid Razzak, Sameen Siddiqi

**Affiliations:** 1 Department of Emergency Medicine, Aga Khan University, Karachi, Pakistan; 2 Department of Community Health Sciences, Aga Khan University, Karachi, Pakistan; 3 Department of Emergency Medicine, Johns Hopkins School of Medicine, Baltimore, USA

**Keywords:** Trauma care system, Afghanistan, Emergency care system, Perceptions, Mixed method, Healthcare professionals

## Abstract

**Background:**

Trauma and injury contribute to 11% of the all-cause mortality in Afghanistan. The study aimed to explore the perceptions of the healthcare providers (pre and in-hospital), hospital managers and policy makers of the public and private health sectors to identify the challenges in the provision of an effective trauma care in Kabul, Afghanistan.

**Methods:**

A concurrent mixed method design was used, including key-informant interviews (healthcare providers, hospital managers and policy makers) of the trauma care system (*N* = 18) and simultaneous structured emergency care system assessment questionnaire (*N* = 35) from July 15 to September 25, 2019. Interviews were analyzed using content analysis approach and structured questionnaire data were descriptively analyzed.

**Results:**

Four themes were identified that describe the challenges: 1) pre-hospital care, 2) cohesive trauma management system, 3) physical and human resources and 4) stewardship. Some key challenges were found related to scene and transportation care, in-hospital care and emergency preparedness within the wider trauma care system. Less than 25% of the population is covered by the pre-hospital ambulance system (*n* = 23, 65.7%) and there is no communication process between health care facilities to facilitate transfer (*n* = 28, 80%). Less than 25% of patients with an injury requiring emergent surgery have access to surgical care in a staffed operating theatre within 2 h of injury (*n* = 19, 54.2%) and there is no regular assessment of the ability of the emergency care system to mobilize resources (human and physical) to respond to disasters, and other large-scale emergencies (*n* = 28, 80%).

**Conclusion:**

This study highlighted major challenges in the delivery of trauma care services across Kabul, Afghanistan. Systematic improvement in the workforce training, structural organization of the trauma care system and implementing externally validated clinical guidelines for trauma management could possibly enhance the functions of the existing trauma care services. However, an integrated state-run trauma care system will address the current burden of traumatic injury more effectively within the wider healthcare system of Afghanistan.

## Background

Trauma is a time-dependent health issue that requires an immediate healthcare intervention to reduce the chances of mortality and disability [1]. The major focus of the trauma care is the rapid transportation to the facility where appropriate trauma care should be available and definitive treatment can be delivered within the first hour of the injury [2]. The trauma care system is well institutionalized with the given major operational components: prevention activities, communication structure, medical direction, training of the trauma workforce, pre-hospital care, transportation care, triage, in-hospital care, rehabilitation, public education and evaluation of trauma capacity [3]. Trauma care system encompassing a comprehensive pre-hospital and in-hospital service delivery model has been proved to reduce mortality, morbidity and revamp functional outcomes [4–6].

The system of trauma care in low- and middle-income countries (LMIC’s) come across number of challenges that include accessibility, limited resources and lack of infrastructure [7, 8]. In many settings of the LMICs, the recognition of the impact of trauma on public health is very limited [9] and there is negligible emphasis on public education regarding the prevention of trauma and injury [10, 11]. In addition, rapid urbanization and industrialization in LMICs have shifted the emphasis of the disease burden towards trauma [12]. Evidence shows that 90% of mortality related to trauma turn up in LMICs [13]. This burden demands a public health attention and a well-functioning trauma care system. However, implementation of the trauma care system tends to be highly resource demanding which could be a deterrent factor to implement it in the low resource countries [11].

Afghanistan is a noncoastal country with a population of 37.2 million in 2018 [14]. Afghanistan has been affected by conflicts for three and half decades which has incapacitated the health infrastructures [15]. All over Afghanistan, there are 3135 health facilities including basic health centers, district hospitals, provincial hospitals and specialty hospitals that ensure access to 87% of the population within 2 h distance [16]. In Kabul, there are 31 hospitals including two trauma centers which provide secondary and tertiary healthcare services. In addition, there is only one public ambulance service that provide services as part of the pre-hospital care in Kabul [17]. This public operated ambulance service has fifteen stations across Kabul from where they are operating the ambulances in case of emergencies. There is no fee for services of this publicly owned ambulance provider and its services are widely available to entire population of Kabul on toll-free number. There are 29 ambulances in total to cater the needs of 4.6 million population in Kabul during emergency situations. It is also noteworthy that Afghanistan’s health system is largely dependent on foreign aid and a large portion of health services provisions are contracted out to Non-governmental organizations [18].

Trauma and injury contribute to 11% of the all-cause mortality in Afghanistan [19–21]. Man-made and natural disasters have also contributed to the large pool of trauma-related morbidity and mortality. Evidence indicates that, trauma and injury is the principal cause for people living with disabilities in Afghanistan [14]. Less than 11% of the seriously injured people from the road traffic crashes are transferred to emergency health care centers by ambulances in Afghanistan [22]. Given the long history of conflict in Afghanistan, the system of trauma care is arguably even more important in this context. There is a very little context-relevant guidance available to help the planners of the healthcare system in developing a well-functioned trauma care system. A perception-based assessment of the pertinent key-informants can buy-in for the effective implementation of the trauma care system in Afghanistan.

### Aim of the study

This study aimed to explore the perceptions of the healthcare providers (pre and in-hospital), hospital managers and policy makers of the public and private health sectors to identify the challenges in the provision of an effective trauma and emergency care in Kabul, Afghanistan.

## Methods

### Design

A concurrent mixed-method approach was utilized to capture the contextual information based on the perceptions of the healthcare providers (pre and in-hospital), hospital managers and policy makers. Qualitative and quantitative data were collected and analyzed at the same time in a single phase. This study was conducted in Kabul, Afghanistan.

### Study setting

Healthcare system in Afghanistan is managed by the Ministry of Public Health (MoPH) and has provincial departments all over 34 provinces. Formulation of health policies, mobilization of resources and regulation of health sector is centrally governed. Healthcare including acute trauma care is provided by a mix of public and private providers.

Kabul is the capital and the largest province of Afghanistan with a population of 4.63 million. Administratively, Kabul is divided into 17 districts. In Kabul, there are 31 hospitals providing secondary and tertiary healthcare services. There is only one public ambulance service namely, the Kabul Ambulance Service that provide services as part of the pre-hospital care in Kabul. There is also a private ambulance system which is based on fee for service.

### Sampling and recruitment of key informants

The sample pool comprised of distinctive key-informants, including pre-hospital administrator, specialist cadres, emergency doctors and nurses. Potential key informants were recruited with the help of consultation with the General Directorate of Curative Medicine at MoPH. The selection of respondents was ensured by including a wide range of participants from both hospital settings and ministry of public health. Purposive sampling technique was used in which all the key informants have become the part of the study after meeting the enrollment criteria; minimum 2 years of experience in provision of trauma care for being enough acquainted with system of trauma care in Kabul.

### Interviews and data collection

Two specific tools were used for collecting data for the purpose of the study. The first tool was an interview guide which was internally developed with the help of a literature review [23] and discussion with subject experts for in-depth interviews (Added as supplementary file 1). The interview guide focused on three main areas: 1) assessing broad understanding of trauma care system, 2) challenges present in the current trauma care pathway and 3) recommendations to improve the current trauma care system. The second tool was developed by World Health Organization (WHO) for assessing emergency care system. The emergency care system assessment (ECSA) tool was then adapted for local use in Afghanistan by narrowing its focus to scene and transportation care, in-hospital care and emergency preparedness. The source of this tool is department of emergency and trauma care, WHO Geneva (Added as supplementary file 2).

The data was collected from July 15 to August 26, 2020 through face to face interviews which was conducted individually with each key informant to lessen the probability of acquiescence and habituation bias. The duration of interview ranged from 25 to 40 min. The first author (UK) interviewed the key informants at their respective workplaces. The qualitative data were collected from the key-informant until redundancy occurred in their responses and saturation was achieved.

### Data analysis

**Fig. 1:**
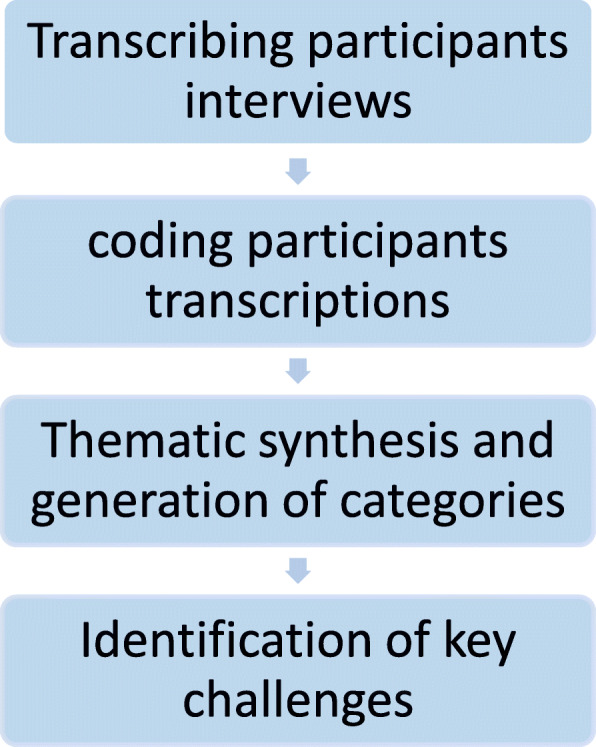
Qualitative data collection and analysis sequence

For qualitative analysis a research assistant transcribed all the interviews in local language (Persian) which were then translated to English language by another research assistant (Fig. 1). These translated transcriptions were then re-translated to Persian language to reduce the effect of lost in translation and interpretation bias. The inconsistencies in the translation were tidied up to avoid the misleading analysis. NVivo version 11 was used for qualitative analysis. A content analysis approach was applied to identify the emerging themes and sub-themes [24]. Rigor and trustworthiness in the study was established through following Lincoln and Guba’s criteria [25]. Credibility of the study was enhanced by emphasizing the aim to learn from respondents through an open and non-judgmental attitude of the interviewer during the key-informant interviews. The content of the transcripts was interpreted by two authors (UK & AA) independently that further contributed to the credibility of the study. In addition, data gathered from the ECSA tool were entered in the SPSS Version 25.0 (SPSS Inc., Chicago, Ill., USA). Descriptive frequencies were computed for the variable of interests.

### Ethical considerations

The study was approved by the institutional review board (IRB) of the Afghanistan National Institute of Public Health (reference number: NS.0619.0032) and the ethical review committee of the Aga Khan University in Karachi, Pakistan (reference number: 2019–1452-4210). Written informed consent was obtained from all the participants at the beginning of an interview and permission was sought for recording the interview. The confidentiality of the respondents was maintained throughout the study and anonymity was ensured by using unique identification numbers for each participant.

## Results

### Demographic characteristics of the interviewees

Interviews were conducted with 18 key-informants from wide range of health services including ministry of health, public and private health facilities and pre-hospital providers. Characteristics of the interviewees are summarized in Table 1. Of the 18 interviewees, most of them were male (*n* = 15, 83.4%) and majority of the participants (*n* = 8, 44.4%) had 8–10 year of experience dealing with trauma and emergency care in Kabul, Afghanistan.

**Table 1:** Characteristics of the interviewees (*N* = 18)

Characteristics	n (%)
**Age**	
30–35 years	3 (16.6)
36–40 years	10 (55.5)
41–45 years	2 (11.1)
46–50 years	3 (16.6)
**Gender**	
Male	15 (83.4)
Female	3 (16.6)
**Respondent’s Type of Institution**	
Public	11(61.2)
Private	7 (38.8)
**Years of Experience**	
2–4 years	2 (11.1)
5–7 years	5 (27.8)
8–10 years	8 (44.4)
11–13 years	3 (16.6)
**Primary role of the Respondents**	
Managers in the Ministry of Public Health	2 (11.1)
Hospital Managers	4 (22.2)
Physicians	4 (22.2)
Nurses	4 (22.2)
Ambulance Staff	3 (16.6)
Ambulance Administrator	1 (5.5)

### Demographic characteristics of the ECSA respondents

A total of 35 respondents completed the structured questionnaire. Most of the respondents were male (*n* = 26, 74.2%) and had been involved for in managing trauma and emergency care for 5–7 years. The characteristics of the respondents are given in Table 2. Majority of the respondents identified themselves as clinical provider (*n* = 20, 57.1%).

**Table 2:** Characteristics of the ECSA respondents (*N* = 35)

Characteristics	n (%)
**Age**	
30–35 years	4 (11.4)
36–40 years	7 (20)
41–45 years	19 (54.2)
46–50 years	5 (14.4)
**Gender**	
Male	26 (74.2)
Female	9 (25.8)
**Respondent’s Type of Institution**	
Public	21 (60)
Private	14 (40)
**Years of Experience**	
2–4 years	13 (37.5)
5–7 years	16 (45.4)
8–10 years	4 (11.4)
11–13 years	2 (5.7)
**Primary role of the Respondents**	
Policy maker	2 (5.7)
Pre-hospital Administrator	3 (8.6)
Head of surgery, trauma or emergency unit	9 (25.7)
Researcher or epidemiologist	1 (2.9)
Clinical provider	20 (57.1)

An analytical thematic framework was developed by identifying the emerging themes from the transcribed interviews. Four key themes were synthesized: 1) pre-hospital care, 2) cohesive trauma management system, 3) physical and human resources and 4) stewardship. These themes are further categorized into sub-themes which were ascertained by grouping the related phrases from the interview transcripts. Table 3 shows the themes and sub-themes in the form of thematic analytical framework.

**Table 3:** Analytical themes and sub-themes based on participant’s perceptions

Themes	Sub-themes
Pre-hospital care	Ambulances
	Layman involvement
	Transportation care
	Road infrastructure
	Universal access number
Cohesive trauma management system	Multidisciplinary approach
	Implementing trauma care guidelines
Physical and human resources	Trauma care workforce
	Physical equipment/supplies
	Technical capacity
Stewardship	Accountability
	Quality improvement approaches

#### Pre-hospital care

The participants expressed concerns related to the provision of trauma care at the pre-hospital level. Among the many factors hindering the delivery of effective pre-hospital trauma care; inadequate ambulances, bystander involvement, poor road infrastructure and lack of universal access number were highly emphasized.

Many participants talked about the challenges related to the availability, functions and transportation mechanism of the ambulances. The number of ambulances to cater the needs of trauma victims in Kabul is inadequate and the ambulances lack adherence to appropriate medical direction protocols for transportation and transfer. Furthermore, perpetuating environmental factors, sub-standard road infrastructures and untrained bystander involvement in medical care complicates the management of trauma care at the pre-hospital level.

*“These ambulances are meant to transport the patients from the scene to the hospital…there is no medical care available during transportation”. (Participant 9)*

*“Just because there are no protocols to manage the transportation care and transfer… there have been many instances in the past when critical patients have been taken to the low-resourced hospitals… and the management of the critically injured patients have been affected” (Participant 11)*

Other challenge related to the optimal pre-hospital care was sub-standard road infrastructure. It causes delay in response time for ambulances.

*“Nearly 30% of the roads in Kabul are not constructed for example interior Qargha (place in north of Kabul) …now if there is an emergency case in this area…it is very difficult for ambulance to reach there in less time” (Participant 4)*

The participants also expressed that majority of the injured patients are transported either by family members, community residents or bystanders. These individuals are untrained and may increase the complications. However, in the absence of immediate pre-hospital care, these individuals can be a good source in transporting the patients to the healthcare facility.

*“In most of the cases…such as road traffic injuries and mass casualties, the injured victims are transported by the taxi drivers, and bystanders…they are unskilled and try to help with inappropriate interventions.” (Participant 7)*

*“Most of the time, taxi drivers, community residents, and family members take most of the injured patients to the hospital in Kabul.” (Participant 9)*

#### Cohesive trauma management system

The participants described that systems for trauma management in Kabul needs multidimensional functions. The current trauma care approach is uncoordinated and complicated in terms of navigating the appropriate trauma services. Few participants expressed that trauma care becomes challenging when there is a gap in implementing guidelines and protocols both at the pre-hospital and in-hospital level. In addition, the receiving hospitals needs to be well-resourced with the essential supplies and workforce to provide the optimal level of care to the trauma patients.

*“There is no well-articulated communication system that should respond in the emergency conditions and notify all the hospitals to be prepared to deal with the mass casualties.” (Participant 5)*

*“There are number of hospitals with well-established emergency rooms…however, they are not adhering to some standard protocols for the management of trauma.” (Participant 9)*

Participants also talked about poor interagency coordination at the pre-hospital level, specifically during large-scale emergencies that hinders the rapid evacuation and optimal trauma care.

*“Some of the major challenges that we face is coordinating with the police when they put the cordon at the site of blast and do not allow our ambulances to get into the site of explosion which obviously cause delay in the care of those who have got massive bleeding and need immediate care” (Participant 9)*

Participants described some other challenges regarding the management of trauma patients in the emergency rooms of the hospitals including unclear roles of the healthcare professionals that often creates confusion and chaotic situation. There is a need of a trauma team in the hospitals with pre-defined roles and responsibilities.

*“We need a team of trauma care…that should have competent doctors, nurses and paramedics to deal with the emergency situations” (Participant 2)*

*“We have people working in the emergency unit with undefined roles…the situation of managing critical trauma care often creates confusion and anxiety…with this kind of disorganized care…I believe we would rather endanger the patient’s life”. (Participant 17)*

#### Physical and human resources

Participants mentioned inaccessibility to appropriate physical and human resources an important factor for ineffective trauma care. Most of the hospitals in Kabul designated for trauma care lack trauma workforce. Furthermore, participants expressed that these hospitals are also deficient in physical resources such as resuscitation equipment to manage critical emergencies.

*“The administration in most of the hospitals is such…that patient’s families have to bring the supplementary supplies when there is some surgery planned” (Participant 17)*

*“Most of the emergency departments don’t even have the crash cart for emergency situations” (Participant 5)*

Some participants mentioned the need for diagnostic equipment such as radiological investigating machines to initiate the appropriate treatment. Additionally, some participants reported that hospitals lack technical professionals to fix the diagnostic machines.

*“We do not have Computed tomography Scan and Magnetic Resonance Imaging (MRI) machines in many hospitals…I believe they are very important in some cases” (Participant 13)*

“*We don’t have technical people who can fix the machines used in hospital like biomedical engineers” (Participant 11)*

Some other challenges were inappropriate staffing in the hospitals and lack of trauma care training as described by the participants

*“It is very surprising to tell you…that midwives are deployed in the burn ward” (Participant 3)*

*“The healthcare providers in the emergency room are not trained enough to deal with the critical trauma patients.” (Participant 1)*

#### Stewardship

According to the participants, the health authorities lack a unified vision to deal with trauma emergencies. There is a gap in the current trauma care system of Afghanistan in terms of having interagency strategic plan, quality improvement approaches and appropriate assignment of trauma related tasks.

**“***The emergency department of the public hospitals are funded by an external agency that functions completely independent of the hospital structure.” (Participant 4)*

*“No one asks about the quality of care…ministry of health should have some mechanism to assess the quality of care.” (Participant 16)*

Lack of accountability and unresponsive to the monetary motivation of the trauma care workforce were some other challenges mentioned by the participants that affect the quality of trauma care.

*“Many nurses who are employed in these hospitals have dual job…because they are not paid enough to run their livelihoods.” (Participant 10)*

### WHO trauma and emergency care system assessment outcomes

A total of 35 mixed healthcare professionals responded to the WHO ECSA survey instrument. The primary role of these respondents ranged from pre-hospital provider, head of surgery or emergency unit, clinical provider, epidemiologist to policy managers. The following sections summarizes the perspectives of these respondents in response to emergency and trauma care system functionality.

### Scene care and transportation

Respondents reported that population of Kabul is partially covered through emergency care access number (Kabul ambulance services-102). Less than 25% of the population is covered through this ambulance system. In addition, the coverage in rural areas is extremely low. Table 4 shows the view of respondents regarding scene care and transportation. Currently, the pre-hospital care is not governed through any kind of system-wide protocols. Participants also deemed the need for communication system to provide on-scene clinical guidance. Respondents felt that existing number of ambulances is inadequate to cater the needs of population. Furthermore, there is no policy to ensure that ambulance service providers have adequate equipment in ambulances to manage patients. Additionally, there is no systematic process of communication for healthcare facilities to assist them with transfer information.

### In-hospital trauma care and emergency preparedness

Respondents reported that less than 25% of the population have access to a well-equipped 24 h in-hospital trauma and emergency care. Table 5 shows respondents view regarding in-hospital trauma care and emergency preparedness. Condition-specific protocols for emergency conditions are not consistent and their use is also not assured. Moreover, less than 25% of the patients who require immediate surgical intervention have access to surgical care in a staffed operating theatre within 2 h. Approximately 25–50% of the facilities dealing with trauma emergencies have triage protocol. The Emergency Severity Index (ESI) algorithm is widely used by many hospitals for triaging. Emergency preparedness across Afghanistan is coordinated by National Command Control Center for Emergency. There is an emergency response plan, but it lacks interagency coordination. The capacity of emergency care system to respond to large scale emergencies is seldom assessed and disaster drills are reasonably infrequent.

**Table 4:** Respondents views regarding scene care, transport and transfer

Indicators	***N*** = 35	
	n	%
There are one or more emergency care access number with partial Kabul coverage.	27	77.1
Pre-hospital care is not governed by any system-wide protocols. However, an advisory service (e.g. staffed telephone) may be available for advice regarding pre-hospital care on ad-hoc basis	28	80
There is no communication system that allows on-scene clinical advising from facilities or dispatch centers	26	74.3
There is no system for determining the most appropriate destination for a given patient	29	82.9
Less than 25% of the population is covered by the pre-hospital ambulance system	23	65.7
The number of ambulances is grossly inadequate for the needs of the population	26	74.3
There is no policy to ensure that pre-hospital providers have adequate equipment to care for patients at the scene and during transport	29	83
There is no communication process between health care facilities to facilitate transfer	28	80.0

**Table 5:** Respondents view regarding In-hospital trauma care and emergency preparedness

Indicators	***N*** = 35	
	n	%
Less than 25% of the population have access to 24-h facility-based emergency care	28	80
Some emergency units have protocols to govern key emergency conditions, but these are not consistently used	25	71.4
Less than 25% of patients with an injury requiring emergent surgery have access to surgical care in a staffed operating theatre within 2 h of injury	19	54.2
25–50% of the trauma facilities have triage protocol with designated triage personnel	30	85.7
There is no regular assessment of the ability of the emergency care system to mobilize resources (human and physical) to respond to disasters, and other large-scale emergencies	28	80
There is emergency response plan, but it was created only by one agency, and not in conjunction with other necessary agencies.	26	74.3
There is no system-level plan in place for extraordinary events that specifically identifies a source for additional human resource and alternate transportation mechanism	29	83

## Discussion

This study identifies several challenges and strength in the current trauma care system of Kabul, Afghanistan and gives a comprehensive understanding of the overall trauma and emergency care service delivery. Four key themes were identified: 1) pre-hospital care, 2) cohesive trauma management system, 3) human and physical resources and 4) stewardship. Some of the key obstacles reported were related to scene and transportation care, in-hospital care and emergency preparedness within the wider trauma care system. Despite this, there were some strengthening factors; such as an Italian non-governmental hospital designed for trauma services exclusively for the victims of war. This hospital receives patients from Kabul as well as remote areas of Afghanistan. Its services are free of charge. Moreover, the emergency departments of the few public hospitals are functioning under the “Intensive Care Unit (ICU) project” funded by external donor agencies. The essential equipment is available in the emergency rooms of these hospitals, but the utilization of these equipment is not ensured widely.

The emergency care system assessment (ECSA) tool can be useful in providing a comprehensive picture of the emergency care services in developing countries. It is designed to assess the various components of the emergency care system including governance, financing, emergency care data, pre-hospital care, in-hospital care, emergency preparedness, quality improvement and rehabilitation. However, this tool demands the perceptions of wide range of professionals including policymakers dealing with the emergency care system to present the average situation across the country.

There is only one pre-hospital provider in Kabul to cater the emergency needs of the 4.6 million population which is governed by the MoPH. This public owned pre-hospital provider has fifteen stations in different regions of the Kabul. The total number of ambulances are 29 which is inadequate to meet the population’s emergency needs. Shortage of ambulances is one of the barriers for ineffective pre-hospital trauma care [26, 27]. Conversely, there are private ambulances linked with private hospitals in Kabul, but they are not widely standardized and regulated. Their services are limited to transportation from one facility to another facility in case of referral and transporting patient to home on patient’s preferences.

The current pre-hospital care system in Kabul lacks protocol for triage of the acutely injured patients that impacts the outcome of care negatively. Triage to a non-trauma center increases the mortality rate up to 30% in the initial 2 days for acutely injured patients [28] . Similarly, no system of pre-arrival notification exists between ambulance crew and receiving hospital. Presence of pre-arrival notification communicating severity of injuries, clinical condition of injured patient mechanism of injury, prehospital intervention and estimated time of arrival can significantly enhance preparation at the facility for optimal trauma care [29, 30]. Likewise, there is no medical direction (clinical advising) from dispatch center or trauma facilities to support the trauma care at the scene level and transportation. The outcome of the trauma care at the level of scene and transportation is widely based on the knowledge and skills of the pre-hospital ambulance staff. In addition, inadequate equipment in the ambulances is another barrier to ineffective pre-hospital care. A study conducted in Pakistan, showed that availability of the equipment in ambulances increases the chance of survival for trauma patients [31]. However, the availability of the equipment in the ambulance does not exclusively decide the chance of survival but skills of the pre-hospital care provider plays an equal role where most of the ambulance staffs in Kabul were found not having the necessary training.

The study also found that bystanders play an important role in providing care at the scene and during transportation to the injured. Since bystanders witness many pre-hospital emergencies; a bystander trauma care training that is context appropriate may improve the initial care at the site of injury until emergency medical service is arrived [32]. Poor terrains and narrow roads were found to be some of the barriers for the ambulance providers for transporting the patients timely. A study conducted in Iran to identify the barriers of the pre-hospital trauma care also found that sub-standard road infrastructure impedes the transportation care [26]. Furthermore, there is a poor interagency coordination while responding to the large-scale emergency crisis such as bomb explosion or earthquake etc. The lack of interagency coordination leads to inappropriate mobilization of resources and delayed evacuations. Most of the pre-hospital services in LMIC’s require coordination among the existing pre-hospital agencies for ensuring comprehensive input [33].

The in-hospital trauma care provided is not adequately integrated that negatively impacts the management of trauma patients. A basic training on the most effective and widely accepted approach to initial evaluation of a trauma patients (such as airway, breathing, circulation, disability, exposure) can help emergency and trauma care providers identify and treat most life-threatening conditions [34]. The roles of the different health cadres involved in trauma management are not clear and that creates confusion. The optimal trauma care needs coordination of multispecialty services in the hospital with well-defined roles that collectively makes up a trauma team. This multispecialty approach to trauma care ensures the effective integration of resources and knowledge across the continuum of care has been shown to improve outcomes [4, 35–38]. However, training of the healthcare workforce designated for the management of trauma patients is also a major concern since most of the health care providers were not certified in any trauma related courses such as advanced trauma life support (ATLS). A study conducted in Iran showed a remarkable improvement in the trauma management of the injured patients after providing training to the existing cadres of the trauma care [39]. Training is a critical component of a continuous performance improvement cycle, ensuring that advances in knowledge are translated into practice in a timely manner. Successful training requires the development of and adherence to training standards. The healthcare personnel of the trauma and emergency management in Kabul, Afghanistan can be trained through numerous courses designed to address the burden of trauma comprehensively including Basic Endovascular Skills for Trauma (BEST) and Advanced Trauma Operative Management (ATOM) courses [40]. Furthermore, the lack of clinical protocols and equipment for diagnosis and management of trauma patients was identified a barrier to effective trauma management which is consistent with other LMIC’s [41–44]. Moreover, the equipment to manage resuscitation followed by the lack of physical resources such as imaging technologies are some of the barriers to providing effective trauma care. Similar challenges were reported in other studies from LMIC’s [42, 45, 46]. Subsequently, the assessment of the hospital capabilities for trauma care is infrequent which is supported by the qualitative analysis that ministry of health lacks technical capacity. Many studies have emphasized the assessment of the trauma care capacities by adding quality improvement programs to strengthen the in-hospital trauma care [47, 48]. This study determined these challenges in the provision of trauma care services in Kabul which is the most developed province of Afghanistan and by inferencing; this situation is likely to be worse in other provinces of Afghanistan.

Stewardship has a strategic role in creating a well-functioned and effective trauma care system that addresses the need of injured at all tiers of the care [49]. In the current trauma care system, the accountability and ownership are not clearly defined resulting in compromised care of the wounded. Key decision makers often lack the knowledge, skills, clarity of roles and responsibility, and perspective to address problems in the trauma care system. Furthermore, the trauma care system is part of the overall healthcare delivery system and works best when it is integrated across various components of health system to ensure smooth transition of care. Such system will also create a halo effect for other emergent and time-sensitive conditions and provides necessary support for mass casualty events and disasters [4].

Some strategies are recommended to address the challenges of the trauma care system in Kabul, Afghanistan based on the study’s discussion and analysis of findings. Table 6 gives a description of these strategies.

**Table 6:** Strategies to address the challenges of trauma care system in Kabul

Description of strategies	
1. First aid training of the lay responders including taxi drivers and law enforcing agents	
2. Training of the emergency medical technicians in ambulances	
3. Set up a prehospital emergency response system with a universal access number, trained emergency medical technicians/paramedics and increase the number of appropriately-equipped ambulances up to forty-three ambulances to meet the population demand	
4. Improve system-wide coordination mechanisms	
5. Improve the accessibility and quality of ambulance services through public-private partnership	
6. Mandating trauma care certification for the emergency care providers	
7. Arranging quarterly mock drills of the emergency preparedness plan	
8. Developing trauma care registry to improve medical care for trauma patients	

### Limitations

This study has some limitations, the most important being selection bias. Respondents who agreed to participate in the study may have negative perceptions about the existing trauma care system. This issue was mitigated by recruiting wide range of participants from multiple segments of the healthcare system (MoPH, pre-hospital service provider, emergency response center, public and private hospitals). In addition, the perceptions of the healthcare professionals from an NGO based trauma facility couldn’t have been explored and this may affect our findings on the capacity of trauma care. Furthermore, focus group discussions were not conducted that may have added richness to qualitative responses. However, responses were clustered around the themes presented and no response was omitted from being presented in the results. Despite these limitations, this study provided contextual knowledge regarding the existing status of trauma and emergency care in Kabul, Afghanistan. The qualitative research helped examining the situation in depth which were not previously known and identified number of challenges that impedes the delivery of trauma care effectively. Additionally, a quantitative structured tool was used to estimate the view of respondents and both were triangulated; qualitative and quantitative responses to ensure validity.

## Conclusion

This study highlighted major challenges in the delivery of trauma care services across Kabul, Afghanistan. These are inevitable issues to overcome; such as pre-hospital care, multidisciplinary approach, accountability of health authorities and human and physical resource at pre-hospital and in-hospital settings. Systematic improvement in the workforce training, structural organization of the trauma system and implementing externally validated clinical guidelines for trauma management could possibly enhance the functions of the existing trauma care services. However, an integrated state-run trauma care system will address the current burden of traumatic injury more effectively within the wider healthcare system of Afghanistan.

## Supplementary Information

**Additional file 1.** Interview Guide.

**Additional file 2.** ECSA Structured Questionnaire.

## Data Availability

The data is stored in the repository of the Aga Khan University. The datasets used and analyzed during the current study are available from the corresponding author on reasonable request.

## Abbreviations

LMIC’s: Low- and Middle-Income Countries
MoPH: Ministry of Public Health
WHO: World Health Organization
IRB: Institutional Review Board
ECSA: Emergency Care System Assessment
ESI: Emergency Severity Index
ICU: Intensive Care Unit
ATLS: Advanced Trauma Life Support
BEST: Basic Endovascular Skills for Trauma
ATOM: Advanced Trauma Operative Management
NGO: Non-governmental organization
