# Supplementary material for: Perception of the healthcare professionals towards the current trauma and emergency care system in Kabul, Afghanistan: a mixed method study
Source: BMC Health Serv Res. 2020 Oct 29;20:991. doi: 10.1186/s12913-020-05845-8 (PMC7596957; doi:10.1186/s12913-020-05845-8)
Supplement: Supplementary file 1 — Additional file 1. Interview Guide. [file 12913_2020_5845_MOESM1_ESM.doc]

**“Assessment of the Pre-hospital and Facility-based Trauma Care”**

Interview Guide

*Interviewer introduces himself and the project and inform them about the interview (also ensure that they are in a safe and secure place and comfortable)*

*Informed consent obtained as per rules of ERC*

*Permission to record/tape interview*

*Note to interviewer: these questions are meant as a guide for your interview; allow the interviewee time and space to express themselves*

Opening question:

- Get basic information on interviewee such as age, years of experience, education level, designation and affiliation with organization.
- What do you understand by pre-hospital and facility-based trauma care?

How the pre-hospital care system is functioning in Kabul?

Use probes such as:

- Is there any UAN number?
- Is there any dispatch system?
- What is the mode of transportation?
- How long patients take to get to the appropriate trauma facility?

What is the biggest challenge in the pre-hospital care system?

Use probe such as:

- What do you think needs to be done to improve pre-hospital care system?

Do you think ambulance staff is trained to provide care during transportation?

- Yes/No

If YES, then which type of trainings have been done?

- _______________
- _______________
- _______________

Do you think ambulance is well equipped to provide care during transportation?

- Yes/No

If YES, then which type of equipment/supplies/medicines are available in the ambulance?

- _______________
- _______________
- _______________
- _______________

What do you think needs to be done to improve pre-hospital care system?

How the facility-based trauma care system is functioning?

Use probes such as:

- What are the protocols/guidelines followed to provide care to the injured in facility?
- Which surgical interventions are available to provide care to the injured?
- How the is the care coordinated between ambulance and trauma facility?

Which categories of cadres are available in the trauma facility to provide care to the injured?

- _____________
- _____________
- _____________
- _____________
- _____________

Do you think emergency care providers are trained to provide care to the injured at the facility?

- Yes/No

If YES, then which type of trainings have been done?

- _______________
- _______________
- _______________
- _______________

How the facilities are monitored for its optimal performance?

Use probes such as:

- What is the reporting mechanism?
- Are there any quality improvement programs?
- How often the facility is assessed for quality assurance?

What do you think needs to be done to improve facility-based trauma care?
