# Supplementary material for: Perception of the healthcare professionals towards the current trauma and emergency care system in Kabul, Afghanistan: a mixed method study
Source: BMC Health Serv Res. 2020 Oct 29;20:991. doi: 10.1186/s12913-020-05845-8 (PMC7596957; doi:10.1186/s12913-020-05845-8)
Supplement: Supplementary file 2 — Additional file 2. ECSA Structured Questionnaire. [file 12913_2020_5845_MOESM2_ESM.docx]

**World Health Organization**

**Emergency Care System Assessment Tool**

**Assembling the Survey Team**

The WHO emergency care system assessment tool is a structured survey that can be answered in writing or via interviews. In order to ensure an accurate description of emergency care system components, respondents should come from a range of settings and might include policy makers; hospital administrators; heads of nursing, medicine, critical care, or emergency units; emergency care providers; pre-hospital care leaders; and emergency care researchers or epidemiologists.

**Instructions for Respondents to answer the survey questions**

In general, answers should reflect the average situation across a nation, rather than the highest performing example in the system.

The assessment tool is divided into 4 sections:

- Scene Care
- Transport and Transfer
- Facility-based Care
- Emergency Preparedness

The Emergency Care System Assessment Tool contains two different questions formats:

1. Basic fact questions with Yes/No or tick-box answers
2. Roadmap questions where the answers numbered 1 to 5 represent progressive levels of emergency care system development.

Please note that the stepwise progression of the roadmap answer choices is approximate. While 1 represents the most basic conditions, and 5 represents a fully developed emergency care system component, the answers in between may represent similar levels of development because there are multiple possible ways for emergency care system development. For example, a 2 will always reflect a state of lower development than a 5, but 3 and 4 may represent two equally feasible paths for system advancement.

Wherever an answer on a roadmap question falls between two options, select the lower number. In other words, an answer should be selected only when every criterion within the description has been met. For instance, if many, but not all components of answer 4 apply, then chose answer 3 to avoid confusion with in-between answers.

Some questions ask respondents to estimate what proportion of the population has access to a certain service. Exact figures are not needed to respond to these questions, and the answers use approximate terms: *few, some, most* and *all.* In this tool, *few* means less than 25%, *some* means approximately 25-50%, *most* means over 50% and all suggest complete coverage.

# Respondent Information

| 1.1 | **Respondents Name:** |  | |
| --- | --- | --- | --- |
| 1.2 | **What is your primary role in the Kabul’s emergency care system? (Select one)** | | |
| - Policy maker - Pre-hospital Administrator - Facility-based Administrator - Head of Surgery, trauma or emergency Unit | | | - Researcher or epidemiologist - Clinical Provider - Other: Please specify |
| 1.3 | **Select any previous roles you had in the country’s emergency care system:** | | |
| - Policy maker - Pre-hospital Administrator - Facility-based Administrator - Head of Surgery, trauma or emergency Unit | | | - Researcher or epidemiologist - Clinical Provider - Other: Please specify |

# Scene Care

| 2.1 | | | **Is there a legislation requiring fixed and mobile telephone companies to provide free connection to an emergency care access number?**  *Examples of emergency care services access number may include 911 or 112* | | | | | Yes | | | | | | No | | | I don’t know | | | | | | | |
| --- | --- | --- | --- | --- | --- | --- | --- | --- | --- | --- | --- | --- | --- | --- | --- | --- | --- | --- | --- | --- | --- | --- | --- | --- |
| 2.2 | | | **Coverage of emergency care services access number:**  *Examples of emergency care services access number may include 911 or 112*  *(If there is no emergency care services access number, please skip directly to question 2.5)* | | | | | | | | | | | | | | | | | | | | | |
| There is no emergency care access number | | | | | | | | | | | | | | | | | 1 | | | | | | | |
| There are one or more emergency care services access number with partial Kabul coverage overall. Areas of the Kabul remain uncovered. | | | | | | | | | | | | | | | | | 2 | | | | | | | |
| There are multiple emergency care services access number that, taken together, provide total Kabul coverage. No single number cover the whole Kabul but all areas are covered by some number. | | | | | | | | | | | | | | | | | 3 | | | | | | | |
| There is one emergency care services access number with total Kabul coverage, but additional numbers with partial coverage still exist. | | | | | | | | | | | | | | | | | 4 | | | | | | | |
| There one single emergency care services access number with total Kabul coverage. All areas use this number, and no other numbers exist | | | | | | | | | | | | | | | | | 5 | | | | | | | |
| I don’t know | | | | | | | | | | | | | | | | |  | | | | | | | |
| Cannot answer for the reason (Explain): | | | | | | | | | | | | | | | | |  | | | | | | | |
| **Please list the main emergency care services access number(s) used in Kabul:** | | | | | | | | | | | | | | | | | | | | | | | | |
|  | | | | | | | | | | | | | | | | | I don’t know | | | | | | | |
| 2.3 | | | **Estimate the proportion of the population that knows and can properly use an emergency care services access number by memory:**  *Examples of emergency care services access number may include 911 or 112* | | | | | | | | | | | | | |  | | | | | | | |
| None | | | | | | | | | | | | | | | | | 1 | | | | | | | |
| Few (<25%) | | | | | | | | | | | | | | | | | 2 | | | | | | | |
| Some (25-50%) | | | | | | | | | | | | | | | | | 3 | | | | | | | |
| Many (51-75%) | | | | | | | | | | | | | | | | | 4 | | | | | | | |
| Most (76-95%) | | | | | | | | | | | | | | | | | 5 | | | | | | | |
| Almost all (>95%) | | | | | | | | | | | | | | | | | 6 | | | | | | | |
| I don’t know | | | | | | | | | | | | | | | | |  | | | | | | | |
| Cannot answer for the reason (Explain): | | | | | | | | | | | | | | | | |  | | | | | | | |
| 2.4 | | | **If there are emergency care services access number(s), what services are provided?** | | | | | | | | | | | | | | | | | | | | | |
| Dispatch of providers to a scene | | | | | | | | | | Yes | | | | No | | | I don’t know | | | | | | | |
| Basic clinical advice to bystanders | | | | | | | | | |  | | | |  | | |  | | | | | | | |
| Medical direction of pre-hospital providers | | | | | | | | | |  | | | |  | | |  | | | | | | | |
| Information reporting to receiving facility | | | | | | | | | |  | | | |  | | |  | | | | | | | |
| Automated tracking of caller location via phone | | | | | | | | | |  | | | |  | | |  | | | | | | | |
| Other method of identifying caller location | | | | | | | | | |  | | | |  | | |  | | | | | | | |
| - Other (Describe): | | | | | | | | | | | | | | | | | | | | | | | | |
| - There are no emergency care services access number | | | | | | | | | | | | | | | | | | | | | | | | |
| 6.5 | | | **Are there laws to protect bystanders who provide help to the acutely injured person?** | | | | | | | Yes | | | No | | | | | | I don’t know | | | | | |
| 2.6 | | | **Community-based basic first aid training courses for lay-people:** | | | | | | | | | | | | | | | | | | | | | |
| There are no first aid training for lay-people | | | | | | | | | | | | | | | | | | | | | | | |  |
| There are some first aid training courses for lay-people. However, they are not widely available nor are they regulated | | | | | | | | | | | | | | | | | | | | | | | |  |
| First training courses for lay people are widely available and affordable and are usually regulated by some authority | | | | | | | | | | | | | | | | | | | | | | | |  |
| First aid training courses for lay-people are widely available, and are centrally regulated and certified. | | | | | | | | | | | | | | | | | | | | | | | |  |
| I don’t know | | | | | | | | | | | | | | | | | | | | | | | |  |
| Cannot answer for another reason (Explain): | | | | | | | | | | | | | | | | | | | | | | | |  |
| 6.7 | | | **Are there lay-persons formally designated as emergency care providers?**  ***Lay emergency care responders*** *are persons formally trained and systematically organized to address medical emergencies, instead of or in conjunction with, professional emergency care services. Example could include police officers, fire fighters, taxi drivers, and other community leaders who have received clinical training and integrated into the emergency care system.* | | | | | | | Yes | | | | | No | | | | | I don’t know | | | | |
| 2.8 | | | **Targeted training for lay emergency care providers:**  ***Lay emergency care responders*** *are persons formally trained and systematically organized to address medical emergencies, instead of or in conjunction with, professional emergency care services. Example could include police officers, fire fighters, taxi drivers, and other community leaders who have received clinical training and integrated into the emergency care system.* | | | | **General Emergency Care** | | | | | | | | | **Trauma Care** | | | | | | | | |
| No such lay responder training programs exist | | | | | | |  | | | | | | | | |  | | | | | | | | |
| Occasional training is provided by different groups, but there is no coordination between groups. This training is not integrated into formal administrative or credentialing structures. | | | | | | |  | | | | | | | | |  | | | | | | | | |
| Occasional training is provided by different groups and is integrated into formal administrative or credentialing structures. However, there is no overall coordination of efforts or centralized quality control | | | | | | |  | | | | | | | | |  | | | | | | | | |
| Training is conducted regularly and centrally coordinated to assure quality of curriculum, integrated into credentialing structures, and wide availability of the training. | | | | | | |  | | | | | | | | |  | | | | | | | | |
| Training is conducted regularly and centrally coordinated to assure quality of curriculum, integration into credentialing structures, and wide availability of the training. In addition, there is on-going monitoring of the care provided by these lay emergency care responders. | | | | | | |  | | | | | | | | |  | | | | | | | | |
| I don’t know | | | | | | |  | | | | | | | | |  | | | | | | | | |
| Cannot answer for another reason (Explain): | | | | | | |  | | | | | | | | |  | | | | | | | | |
| 2.9 | | | **If there are lay emergency care responders, are they provided with basic emergency providers kits?**  ***Lay emergency care responders*** *are persons formally trained and systematically organized to address medical emergencies, instead of or in conjunction with, professional emergency care services. Example could include police officers, fire fighters, taxi drivers, and other community leaders who have received clinical training and integrated into the emergency care system.* | | | | | | Yes | | No | | | | | | | I don’t know | | | | | | |
| - There are no lay emergency care responders | | | | | | | | | | | | | | | | | | | | | | | | |
| 2.10 | | | **Are there personnel trained and certified specifically as ambulance providers?** | | | | | | Yes | | No | | | | | | | I don’t know | | | | | | |
| 2.11 | | | **Initial certification for ambulance providers:** | | | | | | | | | | | | | | | | | | | | | |
| There is no process of certifying ambulance providers | | | | | | | | | | | | | | | | | | | | | | 1 | | |
| There are multiple certification processes for ambulance providers; however certification is not required in all cases to practice. | | | | | | | | | | | | | | | | | | | | | | 2 | | |
| There are multiple certification processes for ambulance providers and some certification is required in all cases to practice. | | | | | | | | | | | | | | | | | | | | | | 3 | | |
| There is a centralized national certification process for ambulance providers, but there are still areas where this certification in not required to practice | | | | | | | | | | | | | | | | | | | | | | 4 | | |
| There is a centralized national certification process. Certification is required in all cases to practice. | | | | | | | | | | | | | | | | | | | | | | 5 | | |
| I don’t know | | | | | | | | | | | | | | | | | | | | | |  | | |
| Cannot answer for another reason (Explain): | | | | | | | | | | | | | | | | | | | | | |  | | |
| 2.12 | | | **Dispatch of pre-hospital providers:**  ***Pre-hospital providers*** *may refer to both formally trained lay emergency care responders as defined above or certified* ***ambulance providers*** | | | | | | | | | | | | | | | | | | | | | |
| There is no method for dispatch of pre-hospital providers | | | | | | | | | | | | | | | | | | | | | | 1 | | |
| Dispatch of pre-hospital providers can be done by telephone, radio, or other communication, but is not available system-wide or coordinated centrally | | | | | | | | | | | | | | | | | | | | | | 2 | | |
| Dispatch of pre-hospital providers can be done by telephone, radio, or other communication, and is available system-wide, but is not coordinated centrally | | | | | | | | | | | | | | | | | | | | | | 3 | | |
| Dispatch of pre-hospital providers is available system-wide and coordinated centrally | | | | | | | | | | | | | | | | | | | | | | 4 | | |
| Dispatch of pre-hospital providers is available system-wide and coordinated centrally, and includes the use of automated technology for localization (e.g. identifying injured person’s location, identifying closest responders, generating driver direction, etc.) | | | | | | | | | | | | | | | | | | | | | | 5 | | |
| I don’t know | | | | | | | | | | | | | | | | | | | | | |  | | |
| Cannot answer for another reason (Explain): | | | | | | | | | | | | | | | | | | | | | |  | | |
| 2.13 | | | Standardized pre-hospital care protocols: | | | | | | | | | | | | | | | | | | | | | |
| Pre-hospital care is not governed by any system-wide protocols. There is no supportive clinical advisory services (e.g. staffed telephone) | | | | | | | | | | | | | | | | | | | | | | 1 | | |
| Pre-hospital care is not governed by any system-wide protocols. However, an advisory service (e.g. staffed telephone) may be available for advice regarding pre-hospital care or and ad-hoc basis | | | | | | | | | | | | | | | | | | | | | | 2 | | |
| Some protocol exist to regulate pre-hospital care, however these are not system-wide not reliably monitored. There is no reliable back-up advisory system to provide extra clinical support when needed. | | | | | | | | | | | | | | | | | | | | | | 3 | | |
| System-wide protocols exist to regulate pre-hospital care and are centrally monitored. However there is no reliable back-up advisory system to provide extra clinical support when needed. | | | | | | | | | | | | | | | | | | | | | | 4 | | |
| System-wide protocols exist to regulate pre-hospital care and are centrally monitored. There is also a reliable back-up advisory system to provide extra clinical support where required at all times. | | | | | | | | | | | | | | | | | | | | | | 5 | | |
| I don’t know | | | | | | | | | | | | | | | | | | | | | |  | | |
| Cannot answer for another reason (Explain) | | | | | | | | | | | | | | | | | | | | | |  | | |
| 2.14 | | | **Medical (specialist) guidance of pre-hospital care:**  ***Medical guidance*** *may include specialist input into clinical protocols, clinical practice review, or real time remote clinical advising.* | | | | | | | | | | | | | | | | | | | | | |
| There is no medical guidance to support pre-hospital care | | | | | | | | | | | | | | | | | | | | | | 1 | | |
| There is medical guidance, but it is limited to administrative role and does not address clinical care delivery. | | | | | | | | | | | | | | | | | | | | | | 2 | | |
| There is medical guidance, but it is limited in scope, such as by development of protocols only. There is no component of clinical practice review. | | | | | | | | | | | | | | | | | | | | | | 3 | | |
| There is medical guidance that involves some component of retrospective quality assessment and clinical practice review, but no access to real time support for clinical practice | | | | | | | | | | | | | | | | | | | | | | 4 | | |
| There is medical guidance with active involvement of medical director of the pre-hospital system in development of protocols, access to real-time supervision for difficult cases. | | | | | | | | | | | | | | | | | | | | | | 5 | | |
| I don’t know | | | | | | | | | | | | | | | | | | | | | |  | | |
| Cannot answer for another reason (Explain): | | | | | | | | | | | | | | | | | | | | | |  | | |
| 2.15 | | | | **Communication system that allows on-scene clinical advising from facilities or dispatch centers:** | | | | | | | | | | | | | | | | | | | | |
| There is no such communication system | | | | | | | | | | | | | | | | | | | | | | 1 | | |
| There is a system but it is not technically reliable | | | | | | | | | | | | | | | | | | | | | | 2 | | |
| There is a reliable technical system, however its use is limited as it is not necessarily staffed by advisors at all times | | | | | | | | | | | | | | | | | | | | | | 3 | | |
| There is a reliable technical system staffed by advisors at all times, however use of this system is not regulated by protocols | | | | | | | | | | | | | | | | | | | | | | 4 | | |
| There is a reliable technical system staffed by advisors at all times, and there are clear protocols regulating its use. | | | | | | | | | | | | | | | | | | | | | | 5 | | |
| I don’t know | | | | | | | | | | | | | | | | | | | | | |  | | |
| Cannot answer for another reason (Explain) | | | | | | | | | | | | | | | | | | | | | |  | | |
| 2.16 | **System for determining the most appropriate destination for a given patient:** | | | | | | | | | | | | | | | | | | | | | | | |
| There is no formal pre-hospital system, or there are no destination triage protocols or system. Decisions are made based on provider or patient preference | | | | | | | | | | | | | | | | | | | | | |  | | |
| An advisory service (e.g. staffed telephone) is available for advice regarding patient destination; however there are no protocols governing destination triage. | | | | | | | | | | | | | | | | | | | | | |  | | |
| Some protocols regulate destination triage; however these are not system-wide or reliably monitored. There is not a reliable back-up advisory system to provide clinical support where required. | | | | | | | | | | | | | | | | | | | | | |  | | |
| System-wide protocols regulate destination triage and are centrally monitored. There is not a reliable back-up advisory system to provide clinical support where required | | | | | | | | | | | | | | | | | | | | | |  | | |
| System-wide protocols regulate destination triage and are centrally monitored. There is a reliable back-up advisory system to provide clinical support where required. | | | | | | | | | | | | | | | | | | | | | |  | | |
| I don’t know | | | | | | | | | | | | | | | | | | | | | |  | | |
| Cannot answer for another reason (Explain): | | | | | | | | | | | | | | | | | | | | | |  | | |
| **2.17** | **Estimate the proportion of the population with effective coverage by formal pre-hospital ambulance system (includes care at the scene and during transport):**  ***Effective coverage*** *refers to reliable access to timely on scene emergency/trauma care followed by transport with a provider when needed.*  ***Access*** *to pre-hospital services implies geographic availability, but also includes functional availability (e.g. no financial barriers to access) where a system is unaffordable for a group of people, they should be counted as having no access.* | | | | | | | | | | | | | | | | | | | | | | | |
| None | | | | | | **Urban** | | | | | | **Rural** | | | | | | | | | | | **Total** | |
| Few (<25%) | | | | | |  | | | | | |  | | | | | | | | | | |  | |
| Some (25-50%) | | | | | |  | | | | | |  | | | | | | | | | | |  | |
| Many (51-75%) | | | | | |  | | | | | |  | | | | | | | | | | |  | |
| Most (76-95%) | | | | | |  | | | | | |  | | | | | | | | | | |  | |
| Almost all (>95%) | | | | | |  | | | | | |  | | | | | | | | | | |  | |
| I don’t know | | | | | |  | | | | | |  | | | | | | | | | | |  | |
| Cannot answer for the reason (Explain): | | | | | |  | | | | | |  | | | | | | | | | | |  | |
| 2.18 | | **If known, what is the number of ambulances in Kabul, Afghanistan?** | | | | | | | | | | | | | | | | | | | | | | |
| **Total:** | | | | |  | | | | | | | | | | | | | | | | | | | |
| **Government:** | | | | |  | | | | | | | | | | | | | | | | | | | |
| **Private:** | | | | |  | | | | | | | | | | | | | | | | | | | |
| 2.19 | **If there are regulations and protocols for pre-hospital care, do they govern:** | | | | | | | | | | | | | | | | | | | | | | | |
| All government and private ambulances | | | | | | | | | | | | | | | | | | | | |  | | | |
| Only government ambulances | | | | | | | | | | | | | | | | | | | | |  | | | |
| I don’t know | | | | | | | | | | | | | | | | | | | | |  | | | |
| Cannot answer for another reason (Explain), or comments: | | | | | | | | | | | | | | | | | | | | |  | | | |
| 2.20 | | | **If there is centralized dispatch, does it direct:** | | | | | | | | | | | | | | | | | | | | | |
| All government and private ambulances | | | | | | | | | | | | | | | | | | | | |  | | | |
| Only government ambulances | | | | | | | | | | | | | | | | | | | | |  | | | |
| I don’t know | | | | | | | | | | | | | | | | | | | | |  | | | |
| Cannot answer for another reason (Explain), or comments: | | | | | | | | | | | | | | | | | | | | |  | | | |

# Transport and Transfer

| 3.1 | | | | | **Is there a system of facility designation for trauma and injury related emergencies in Kabul (e.g. trauma centres)?**  ***Designation*** *implies that facilities are accredited against nationally agreed criteria and are assigned a specific level that indicates the capacity of that facility to manage specific needs.* | | Yes | | | | No | | | I don’t know | | |
| --- | --- | --- | --- | --- | --- | --- | --- | --- | --- | --- | --- | --- | --- | --- | --- | --- |
| If so, please list which center designations exist: | | | | | | | | | | | | | | | | |
| 3.2 | | | | | **Transportation to Trauma/medical facilities**  *An* ***ambulance*** *is defined as a vehicle equipped to transfer the acutely injured to a designated care facility. This implies adequate space, functionality, and equipment for care during transport.* | | | Scene to facility | | | | | Inter-facility transfer | | | |
| There are no ambulances or vehicles designated to carry injured patients to medical facilities, and patients and relatives or others must arrange such transfer themselves. | | | | | | | | 1 | | | | | 1 | | | |
| There are no ambulances, but there are designated vehicles to carry patients to medical facilities | | | | | | | | 2 | | | | | 2 | | | |
| There are ambulances to carry patients to medical facilities but the number of ambulances is grossly inadequate for the needs of the population. | | | | | | | | 3 | | | | | 3 | | | |
| There are ambulances to carry patients to medical facilities. Although these resources may be stretched at busy times, they are generally adequate for the needs of the population. | | | | | | | | 4 | | | | | 4 | | | |
| There are always sufficient ambulances to carry patients to medical facilities. | | | | | | | | 5 | | | | | 5 | | | |
| I don’t know | | | | | | | |  | | | | |  | | | |
| Cannot answer for another reason (Explain): | | | | | | | |  | | | | |  | | | |
| 3.3 | Is there a mandatory policy requiring both a driver and a care provider for ambulance transport?  *An* ***ambulance*** *is defined as a vehicle equipped to transfer the acutely injured to a designated care facility. This implies adequate space, functionality, and equipment for care during transport.* | | | | | | | Yes | | No | | | | I don’t know | | |
| 3.4 | Policies to ensure that pre-hospital providers have adequate equipment to care for patients at the scene and during transport | | | | | | | | | | | | | | | |
| There are no regulations or policies about pre-hospital equipment | | | | | | | | | | | | | | | 1 | |
| There are locally-defined regulations and policies about pre-hospital equipment, but they are minimally enforced or enforced in only in few jurisdictions. | | | | | | | | | | | | | | | 2 | |
| There are local (but not national or regional) regulations and policies about pre-hospital equipment, and they are usually enforced. | | | | | | | | | | | | | | | 3 | |
| There are comprehensive national/regional regulations and policies about pre-hospital equipment, meeting most of the criteria laid out in WHO pre-hospital trauma care systems, and they are usually enforced | | | | | | | | | | | | | | | 4 | |
| There are comprehensive national/regional regulations and policies about pre-hospital equipment, meeting most of the criteria laid out in WHO pre-hospital trauma care systems. Compliance with these regulations sis assured by an inspection mechanism and by the lead agency having adequate legal authority to assure compliance. | | | | | | | | | | | | | | | 5 | |
| I don’t know | | | | | | | | | | | | | | |  | |
| Cannot answer for another reason (Explain): | | | | | | | | | | | | | | |  | |
| 3.5 | | **Communication between health care facilities to facilitate transfer** | | | | | | | | | | | | | | |
| there is no process in place for healthcare facilities to communicate with on another regarding transfers. | | | | | | | | | | | | | | | | 1 |
| There is a process of communication between healthcare facilities, but communication about transfer occurs in an uncoordinated manner based on individual decisions | | | | | | | | | | | | | | | | 2 |
| Although a systematic process for healthcare facilities to communicate with one another has been devised, it is infrequently used to facilitate transfers. | | | | | | | | | | | | | | | | 3 |
| There is a systematic process for healthcare facilities to communicate with one another, and this method is frequently used to facilitate transfers. | | | | | | | | | | | | | | | | 4 |
| There is a systematic process for healthcare facilities to communicate with one another regarding transfers; this process is required by protocol and consistently used. | | | | | | | | | | | | | | | | 5 |
| I don’t know | | | | | | | | | | | | | | | |  |
| Cannot answer for another reason (Explain): | | | | | | | | | | | | | | | |  |
| 3.6 | | | | System-wide protocols for inter-facility patient transfers:    ***Transfer criteria*** *govern when patients should be moved between facilities based on clinical condition or availability of diagnostic and management resources.* | | | | | | | | | | | | |
| Transfer criteria are not employed in Kabul. Patients are transferred between healthcare facilities based on individual decisions such as patients or provider preference. | | | | | | | | | | | | | | | | 1 |
| Transfer criteria are employed in Kabul, although not all healthcare facilities use the same criteria. | | | | | | | | | | | | | | | | 2 |
| System-wide transfer criteria exist. However, their usage is not monitored | | | | | | | | | | | | | | | | 3 |
| System-wide transfer criteria exist and are monitored. However, this transfer process is inconsistently employed and transfer decisions are often inappropriate i.e. transferred when not needed, not transferred when needed, or transferred to an inappropriate facility. | | | | | | | | | | | | | | | | 4 |
| System-wide transfer criteria are monitored and consistently shown to ensure that patients are transferred appropriately. | | | | | | | | | | | | | | | | 5 |
| I don’t know | | | | | | | | | | | | | | | |  |
| Cannot answer for another reason (Explain): | | | | | | | | | | | | | | | |  |
| 3.7 | | | **Are there protocols for pre-hospital provider handovers to facilities (i.e. the process required when a pre-hospital provider delivers a patient to a facility)?**  ***Pre-hospital provider*** *may refer to both formally trained* ***lay*** ***emergency care responders*** *as defined above or certified* ***ambulance providers*** | | | Yes | | | No | | | I don’t know | | | | |

# Facility-Based Care

| 4.1 | | | | **If known, what is the number of trauma centers in Kabul, Afghanistan?** | | | | | | | | | | | | | | | | | | |
| --- | --- | --- | --- | --- | --- | --- | --- | --- | --- | --- | --- | --- | --- | --- | --- | --- | --- | --- | --- | --- | --- | --- |
| Total: | | | | |  | | | | | | | | | | | | | | | | | |
| Government: | | | | |  | | | | | | | | | | | | | | | | | |
| Private: | | | | |  | | | | | | | | | | | | | | | | | |
| **If known, what is the number of dedicated emergency units in Kabul, Afghanistan?**  *An* ***emergency unit*** *is any dedicated intake area for acutely ill and injured patients. This may be referred to as the emergency department/room/ward and emergency, casualty, etc.* | | | | | | | | | | | | | | | | | | | | | | |
| Total: | | | | |  | | | | | | | | | | | | | | | | | |
| Government: | | | | |  | | | | | | | | | | | | | | | | | |
| Private: | | | | |  | | | | | | | | | | | | | | | | | |
| **If there is a regional or national poison control center?** | | | | | | | | | | | Yes | | No | | I don’t know | | | | | | | |
|  |  |  |  |  |  |  |  |  |  |  |  | |  | |  | | | | | | | |
| **If yes, is the poison control center available by phone to:** | | | | | | | | | | | Yes | | No | | I don’t know | | | | | | | |
| Clinical providers | | | | | | | | | | |  | |  | |  | | | | | | | |
| General Public | | | | | | | | | | |  | |  | |  | | | | | | | |
| 4.2 | | **Dedicated emergency units responsible for the triage and care of acutely ill or injured patients in facilities:**  *An* ***emergency unit*** *is any dedicated intake area for acutely ill and injured patients. This may be referred to as the emergency department/room/ward and emergency, casualty, etc.*  ***First-level hospitals*** *are lowest level of hospitals also known as district hospitals.*  ***Tertiary Hospitals*** *are highest level of facility with all healthcare services* | | | | | | | | | | | First-level Hospitals | | | | | | Tertiary Hospitals | | | |
| There are no such units | | | | | | | | | | | | | 1 | | | | | | 1 | | | |
| There are emergency units, but they are only accessible for certain hours of the day (not 24 hours per day). Patients are not formally triaged, and are generally seen in order of arrival | | | | | | | | | | | | | 2 | | | | | | 2 | | | |
| There are emergency units that are only accessible for certain hours of the day (not 24 hours per day). During open hours, patients are formally triaged, and are seen in order of acuity. | | | | | | | | | | | | | 3 | | | | | | 3 | | | |
| There are emergency units accessible 24 hours per day, but patients are not formally triaged and are generally seen in order of arrival | | | | | | | | | | | | | 4 | | | | | | 4 | | | |
| There are emergency units accessible 24 hours per day, and patients are formally triaged, and seen in order of acuity. | | | | | | | | | | | | | 5 | | | | | | 5 | | | |
| I don’t know | | | | | | | | | | | | |  | | | | | |  | | | |
| Cannot answer for another reason (Explain) | | | | | | | | | | | | |  | | | | | |  | | | |
| 4.3 | **Emergency unit staffing in facilities:**  *An* ***emergency unit*** *is any dedicated intake area for acutely ill and injured patients. This may be referred to as the emergency department/room/ward and emergency, casualty, etc.*  ***First-level hospitals*** *are lowest level of hospitals also known as district hospitals.*  ***Tertiary Hospitals*** *are highest level of facility with all healthcare services* | | | | | | | | | | | | First-level Hospitals | | | | | | Tertiary Hospitals | | | |
| There are no dedicated emergency units or no providers with specific responsibility for emergency unit patients until they are admitted. | | | | | | | | | | | | | 1 | | | | | | 1 | | | |
| There are staff that register and direct patients in the emergency unit to inpatient areas (the unit has a sorting function, but minimal care is provided). | | | | | | | | | | | | | 2 | | | | | | 2 | | | |
| Providers from inpatient services have on-call responsibility to cover emergency unit patients, but are not assigned to be in the emergency unit. | | | | | | | | | | | | | 3 | | | | | | 3 | | | |
| Providers from inpatient services are assigned to be in the emergency units, rotating through for limited intervals (e.g. 1 month blocks). | | | | | | | | | | | | | 4 | | | | | | 4 | | | |
| There are non-rotating providers that permanently staff the emergency unit. | | | | | | | | | | | | | 5 | | | | | | 5 | | | |
| I don’t know | | | | | | | | | | | | |  | | | | | |  | | | |
| Cannot answer for another reason (Explain): | | | | | | | | | | | | |  | | | | | |  | | | |
| 4.4 | | | **Is there adequate functional equipment necessary for the following in emergency unit at first level hospitals?**  ***First-level hospitals*** *are lowest level of hospitals also known as district hospitals.* | | | None | Few (<25%) | Some  (25-50%) | Many  (51-75%) | | | | Most  (76-95%) | | | Almost all  (>95%) | | | | | | I don’t know |
| Airway management, including intubation | | | | | |  |  |  |  | | | |  | | |  | | | | | |  |
| Breathing intervention, including manual or mechanical ventilation | | | | | |  |  |  |  | | | |  | | |  | | | | | |  |
| Fluid resuscitation | | | | | |  |  |  |  | | | |  | | |  | | | | | |  |
| Vasoactive medications, including at least epinephrine | | | | | |  |  |  |  | | | |  | | |  | | | | | |  |
| Oxygen saturation monitoring | | | | | |  |  |  |  | | | |  | | |  | | | | | |  |
| Cardiac monitoring | | | | | |  |  |  |  | | | |  | | |  | | | | | |  |
| Other (describe): | | | | | | | | | | | | | | | | | | | | | | |
| 4.5 | | | **Is there adequate access to the following diagnostic services in the emergency units at first-level hospitals?**  ***First-level hospitals*** *are lowest level of hospitals also known as district hospitals.* | | | None | Few (<25%) | Some  (25-50%) | Many  (51-75%) | | | | Most  (76-95%) | | | Almost all  (>95%) | | | | | | I don’t know |
| Diagnostic labs | | | | | |  |  |  |  | | | |  | | |  | | | | | |  |
| Radiology | | | | | |  |  |  |  | | | |  | | |  | | | | | |  |
| Other (describe): | | | | | | | | | | | | | | | | | | | | | | |
| 4.6 | | | **Is there adequate functional equipment necessary for the following in emergency unit at tertiary hospitals?**  ***Tertiary Hospitals*** *are highest level of facility with all healthcare services.* | | | None | Few (<25%) | Some  (25-50%) | Many  (51-75%) | | | | Most  (76-95%) | | | Almost all  (>95%) | | | | | | I don’t know |
| Airway management, including intubation | | | | | |  |  |  |  | | | |  | | |  | | | | | |  |
| Breathing intervention, including manual or mechanical ventilation | | | | | |  |  |  |  | | | |  | | |  | | | | | |  |
| Fluid resuscitation | | | | | |  |  |  |  | | | |  | | |  | | | | | |  |
| Vasoactive medications, including at least epinephrine | | | | | |  |  |  |  | | | |  | | |  | | | | | |  |
| Oxygen saturation monitoring | | | | | |  |  |  |  | | | |  | | |  | | | | | |  |
| Cardiac monitoring | | | | | |  |  |  |  | | | |  | | |  | | | | | |  |
| Other (describe): | | | | | | | | | | | | | | | | | | | | | | |
| 4.7 | | | **Is there adequate access to the following diagnostic services in the emergency units at tertiary hospitals?**  ***Tertiary Hospitals*** *are highest level of facility with all healthcare services.* | | | None | Few (<25%) | Some  (25-50%) | Many  (51-75%) | | | | Most  (76-95%) | | | Almost all  (>95%) | | | | | | I don’t know |
| Diagnostic labs | | | | | |  |  |  |  | | | |  | | |  | | | | | |  |
| Radiology | | | | | |  |  |  |  | | | |  | | |  | | | | | |  |
| Other (describe): | | | | | | | | | | | | | | | | | | | | | | |
| 4.8 | | | **Which of the following triage components exist at first-level hospitals?**  ***First-level hospitals*** *are lowest level of hospitals also known as district hospitals.* | | | None | Few (<25%) | Some  (25-50%) | Many  (51-75%) | | | | Most  (76-95%) | | | Almost all  (>95%) | | | | | | I don’t know |
| Formal triage protocols for patients on arrival | | | | | |  |  |  |  | | | |  | | |  | | | | | |  |
| Designated triage personnel at all times | | | | | |  |  |  |  | | | |  | | |  | | | | | |  |
| Time targets for certain triage designations | | | | | |  |  |  |  | | | |  | | |  | | | | | |  |
| Compliance tracking for triage time targets | | | | | |  |  |  |  | | | |  | | |  | | | | | |  |
| Other (describe): | | | | | | | | | | | | | | | | | | | | | | |
| 4.9 | | | **Which of the following triage components exist at tertiary hospitals?**  ***Tertiary Hospitals*** *are highest level of facility with all healthcare services.* | | | None | Few (<25%) | Some  (25-50%) | Many  (51-75%) | | | | Most  (76-95%) | | | Almost all  (>95%) | | | | | | I don’t know |
| Formal triage protocols for patients on arrival | | | | | |  |  |  |  | | | |  | | |  | | | | | |  |
| Designated triage personnel at all times | | | | | |  |  |  |  | | | |  | | |  | | | | | |  |
| Time targets for certain triage designations | | | | | |  |  |  |  | | | |  | | |  | | | | | |  |
| Compliance tracking for triage time targets | | | | | |  |  |  |  | | | |  | | |  | | | | | |  |
| Other (describe): | | | | | | | | | | | | | | | | | | | | | | |
| 4.10 | | | **Are there regulations and/or protocols mandating that acutely ill or injured patients are clinically triaged prior to being required to register?** | | | | | | | | | Yes | | No | | | I don’t know | | | | | |
| 4.11 | | | **Are there emergency/trauma-specific post graduate degree courses for nurses (e.g. a master’s degree or courses in trauma or emergency nursing)?** | | | | | | | | | Yes | | No | | | I don’t know | | | | | |
| 4.12 | | | **Which of the following exist as fully certified specialist or sub-specialist programs that doctors can train for?** | | | | | | | | | Yes | | No | | | I don’t know | | | | | |
| Emergency medicine | | | | | | | | | | | |  | |  | | |  | | | | | |
| Anesthesia | | | | | | | | | | | |  | |  | | |  | | | | | |
| Critical care | | | | | | | | | | | |  | |  | | |  | | | | | |
| Trauma surgery | | | | | | | | | | | |  | |  | | |  | | | | | |
| 4.13 | | | **Specific training in injury/emergency care as part of initial and ongoing certification for providers who regularly care for emergency patients at first-level hospitals:**  ***First-level hospitals*** *are lowest level of hospitals also known as district hospitals.*  ***Mid-level providers*** *are providers other than doctors serving as independent primary providers. Examples may include clinical officer or advanced nurses.*  *Examples of the externally validate courses include advanced cardiac life support (ACLs) or advanced trauma life support (ATLS).* | | | | | | | Nurses | | | | Mid-level providers | | | | | | Doctors | | |
| Providers who regularly care for emergency patients are not required to undergo injury-specific training as part of the initial or on-going certification. | | | | | | | | | | 1 | | | | 1 | | | | | | 1 | | |
| Providers who regularly care for emergency patients must undergo injury-specific training as part of their initial certification, but it is not required for ongoing maintenance of certification. | | | | | | | | | | 2 | | | | 2 | | | | | | 2 | | |
| Providers who regularly care for emergency patients must undergo injury-specific training as part of both initial and on-going certifications. However, compliance with this requirement is low. | | | | | | | | | | 3 | | | | 3 | | | | | | 3 | | |
| Providers who regularly care for emergency patients must undergo injury-specific training as part of both initial and on-going certifications. Such training is readily available and compliance with the requirement is high. However, continuing education courses are rarely externally validated. | | | | | | | | | | 4 | | | | 4 | | | | | | 4 | | |
| Providers who regularly care for emergency patients must undergo injury-specific training as part of both initial and on-going certifications. Such training is readily available and compliance with the requirement is high. Continuing education routinely includes externally validated courses. | | | | | | | | | | 5 | | | | 5 | | | | | | 5 | | |
| I don’t know | | | | | | | | | |  | | | |  | | | | | |  | | |
| Cannot answer for another reason (Explain): | | | | | | | | | |  | | | |  | | | | | |  | | |
| 4.14 | | | **Specific training in injury/emergency care as part of initial and ongoing certification for providers who regularly care for emergency patients at tertiary hospitals**  ***Tertiary Hospitals*** *are highest level of facility with all healthcare services.*  ***Mid-level providers*** *are providers other than doctors serving as independent primary providers. Examples may include clinical officer or advanced nurses.*  *Examples of the externally validate courses include advanced cardiac life support (ACLs) or advanced trauma life support (ATLS).* | | | | | | | Nurses | | | | Mid-level providers | | | | | | Doctors | | |
| Providers who regularly care for emergency patients are not required to undergo injury-specific training as part of the initial or on-going certification. | | | | | | | | | | 1 | | | | 1 | | | | | | 1 | | |
| Providers who regularly care for emergency patients must undergo injury-specific training as part of their initial certification, but it is not required for ongoing maintenance of certification. | | | | | | | | | | 2 | | | | 2 | | | | | | 2 | | |
| Providers who regularly care for emergency patients must undergo injury-specific training as part of both initial and on-going certifications. However, compliance with this requirement is low. | | | | | | | | | | 3 | | | | 3 | | | | | | 3 | | |
| Providers who regularly care for emergency patients must undergo injury-specific training as part of both initial and on-going certifications. Such training is readily available and compliance with the requirement is high. However, continuing education courses are rarely externally validated. | | | | | | | | | | 4 | | | | 4 | | | | | | 4 | | |
| Providers who regularly care for emergency patients must undergo injury-specific training as part of both initial and on-going certifications. Such training is readily available and compliance with the requirement is high. Continuing education routinely includes externally validated courses. | | | | | | | | | | 5 | | | | 5 | | | | | | 5 | | |
| I don’t know | | | | | | | | | |  | | | |  | | | | | |  | | |
| Cannot answer for another reason (Explain): | | | | | | | | | |  | | | |  | | | | | |  | | |
| 4.15 | | | **Estimate the proportion of the population with 24 hour access to facility-based emergency care (in a dedicated unit with independent, non-rotating providers trained in emergency care) without requirement for payment prior to care:** | | | | | | | Urban | | | | Rural | | | | | | Total | | |
| None | | | | | | | | | | 1 | | | | 1 | | | | | | 1 | | |
| Few (<25%) | | | | | | | | | | 2 | | | | 2 | | | | | | 2 | | |
| Some (25-50%) | | | | | | | | | | 3 | | | | 3 | | | | | | 3 | | |
| Many (51-75%) | | | | | | | | | | 4 | | | | 4 | | | | | | 4 | | |
| Most (76-95%) | | | | | | | | | | 5 | | | | 5 | | | | | | 5 | | |
| Almost all (>95%) | | | | | | | | | |  | | | |  | | | | | |  | | |
| I don’t know | | | | | | | | | |  | | | |  | | | | | |  | | |
| Cannot answer for another reason (explain): | | | | | | | | | |  | | | |  | | | | | |  | | |
| 4.16 | | | **Standardized protocols governing management of key emergency conditions:**  ***Externally validated protocols*** *are derived from guidelines such as WHO guidelines for essential trauma care.* | | | | | | | | | | | | | | | | | | | |
| Facility based emergency care is generally not based on protocols. | | | | | | | | | | | | | | | | | | | | | 1 | |
| Some emergency units have protocols, but these are not consistently used or are not externally validated. | | | | | | | | | | | | | | | | | | | | | 2 | |
| Most emergency units use protocols consistently, but these are not standardized or externally validated. | | | | | | | | | | | | | | | | | | | | | 3 | |
| Most emergency units consistently use standardized, externally validated protocols. | | | | | | | | | | | | | | | | | | | | | 4 | |
| All emergency units consistently use externally validated protocols, and compliance with protocols use is tracked. | | | | | | | | | | | | | | | | | | | | | 5 | |
| I don’t know | | | | | | | | | | | | | | | | | | | | |  | |
| Cannot explain for another reason (Explain): | | | | | | | | | | | | | | | | | | | | |  | |
| 4.17 | | | **Is there a protocol for communication with patients about their disposition or discharge?** | | | | | | | | | Yes | | No | | | I don’t know | | | | | |
| 4.18 | | | **Estimated proportion of patients with an injury requiring emergent surgery who have access to surgical care in a staffed operating theatre within two hours of injury:** | | | | | | | | | | | | | | | | | | | |
| None | | | | | | | | | | | | | | | | | | 1 | | | | |
| Few (<25%) | | | | | | | | | | | | | | | | | | 2 | | | | |
| Some (25-50%) | | | | | | | | | | | | | | | | | | 3 | | | | |
| Many (51-75%) | | | | | | | | | | | | | | | | | | 4 | | | | |
| Most (76-95%) | | | | | | | | | | | | | | | | | | 5 | | | | |
| Almost all (>95%) | | | | | | | | | | | | | | | | | | 6 | | | | |
| I don’t know | | | | | | | | | | | | | | | | | |  | | | | |
| Cannot answer for the reason (Explain): | | | | | | | | | | | | | | | | | |  | | | | |
| 4.19 | | | **Is there a national assessment and accreditation scheme for hospitals?**  *Accreditation is based on the capacity of hospitals to care for specific conditions (e.g. injury);* ***assessment and accreditation schemes*** *may use pre-defined and externally validated criteria such as those outlined by the WHO guidelines for essential trauma care.* | | | | | | | General Emergency Care | | | | | | Injury | | | | | | |
| There has been no assessment of the capacity of healthcare facilities to deliver emergency care. | | | | | | | | | | 1 | | | | | | 1 | | | | | | |
| Some health care facilities may be individually assessed on their capacity to deliver emergency care, but this assessment is not uniform throughout Kabul and is not based on a set of externally validated criteria. | | | | | | | | | | 2 | | | | | | 2 | | | | | | |
| All healthcare facilities are assessed on their capacity to deliver emergency care and are nationally accredited, but this assessment is not based on a set of externally validated criteria. | | | | | | | | | | 3 | | | | | | 3 | | | | | | |
| All healthcare facilities are assessed on their capacity to deliver emergency care and are nationally accredited based on a set of externally validated criteria. | | | | | | | | | | 4 | | | | | | 4 | | | | | | |
| All healthcare facilities are assessed on their capacity to deliver emergency care and are nationally accredited based on a set of externally validated criteria. There are mechanisms in place to ensure that this assessment is periodically repeated. | | | | | | | | | | 5 | | | | | | 5 | | | | | | |
| I don’t know | | | | | | | | | |  | | | | | |  | | | | | | |
| Cannot answer for another reason (Explain): | | | | | | | | | |  | | | | | |  | | | | | | |

# Emergency Preparedness

| 5.1 | **Is there regular assessment of the ability of the emergency care system to mobilize resources (human and physical) to respond to disasters, and other large-scale emergencies?** | Yes | No | I don’t know | |
| --- | --- | --- | --- | --- | --- |
| If yes, please describe: | | | | | |
| 5.2 | **Coordinated planning for disasters and other large-scale emergencies:**  *Other* ***necessary agencies*** *may include public health agencies, police, and fire departments, water and sanitation services as well as others.* | | | | |
| There is no emergency response plan | | | | | 1 |
| There is emergency response plan, but it was created only by one agency, and not in conjunction with other necessary agencies. | | | | | 2 |
| There is emergency response plan involving multiple necessary agencies. However, this plan is not centrally coordinated. | | | | | 3 |
| There is emergency response plan involving multiple necessary agencies. This plan is centrally coordinated. However, there is no requirement for periodic evaluation via mock simulation drills. | | | | | 4 |
| There is emergency response plan involving multiple necessary agencies. This plan is centrally coordinated. In addition, there is a requirement for periodic evaluation via mock simulation drills. | | | | | 5 |
| I don’t know | | | | |  |
| Cannot answer for another reason (Explain): | | | | |  |
| 5.3 | **Is there a system-level plan in place for extraordinary events that specifically identifies a source for the following?** | Yes | No | I don’t know | |
| Additional human resource | |  |  |  | |
| Alternate communication strategy | |  |  |  | |
| Additional supplies for mass casualty events | |  |  |  | |
| Additional clinical space for mast casualty events | |  |  |  | |
| Alternate transportation mechanisms for personnel to patients | |  |  |  | |
| 5.4 | **Are facility level plans for extraordinary events required:** | Yes | No | I don’t know | |
| **At first level hospitals?**  ***First-level hospitals*** *are lowest level of hospitals also known as district hospitals.* | |  |  |  | |
| **At Tertiary hospitals?**  ***Tertiary Hospitals*** *are highest level of facility with all healthcare services.* | |  |  |  | |
| 5.5 | **Are there usually facility level security plans in place to protect staff, patients, and infrastructures from violence:** | Yes | No | I don’t know | |
| **At first level hospitals?**  ***First-level hospitals*** *are lowest level of hospitals also known as district hospitals.* | |  |  |  | |
| **At Tertiary hospitals?**  ***Tertiary Hospitals*** *are highest level of facility with all healthcare services.* | |  |  |  | |
| 5.6 | **Are there specific security plans in place to protect staff, patients and infrastructures from violence in the pre-hospital setting?**  ***Pre-hospital*** *includes ambulance services.* | Yes | No | I don’t know | |
